# Relationship between the Biological Clock and Inflammatory Bowel Disease

**DOI:** 10.3390/clockssleep5020021

**Published:** 2023-05-12

**Authors:** Jonathan Giebfried, Axel Lorentz

**Affiliations:** Institute of Nutritional Medicine, University of Hohenheim, Fruwirthstraße 12, 70599 Stuttgart, Germany

**Keywords:** biological clock, circadian rhythm, clock genes, inflammatory bowel disease, intestinal diseases

## Abstract

The biological clock is a molecular oscillator that generates a 24-hour rhythm in accordance with the earth’s rotation. Physiological functions and pathophysiological processes such as inflammatory bowel diseases (IBD) are closely linked to the molecular clock. This review summarizes 14 studies in humans and mice on the interactions between the biological clock and IBD. It provides evidence that IBD negatively affect core clock gene expression, metabolism and immune functions. On the other hand, disruption of the clock promotes inflammation. Overexpression of clock genes can lead to inhibition of inflammatory processes, while silencing of clock genes can lead to irreversible disease activity. In both human and mouse studies, IBD and circadian rhythms have been shown to influence each other. Further research is needed to understand the exact mechanisms and to develop potential rhythm-related therapies to improve IBD.

## 1. Introduction

### 1.1. The Biological Clock

The biological clock is a molecular oscillatory system that is responsible for the rhythmicity of cellular processes [1]. In doing so, it maintains physiological functions and homeostasis of the body. In humans, its rhythm is regularly repeated approximately every 24 h and can be synchronized by environmental factors [2]. For example, light interacts with the central oscillator of the circadian clock located in the suprachiasmatic nucleus (SCN) of the hypothalamus [2]. 

**Figure 1:**
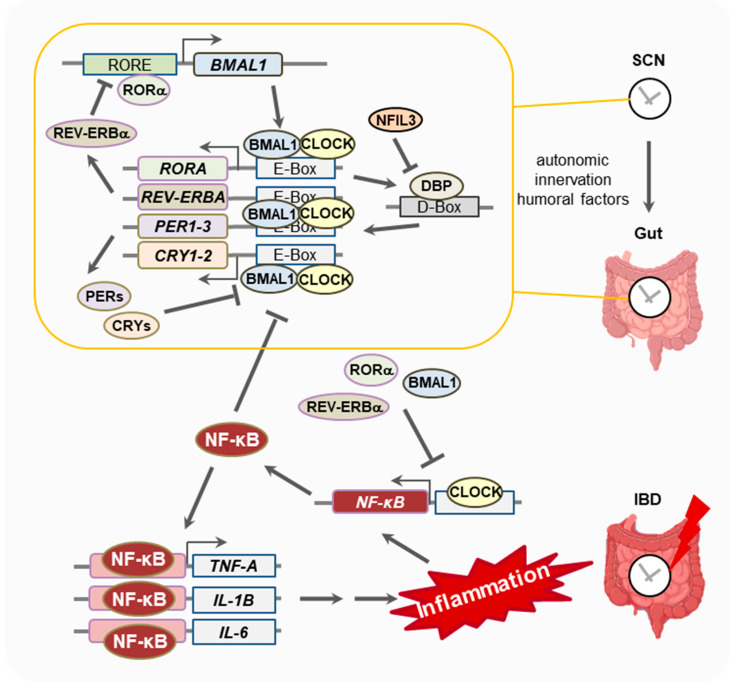
The molecular mechanism of the circadian clock and its potential interaction with inflammation in IBD: The central oscillator of the circadian clock is located in the suprachiasmatic nucleus (SCN) of the hypothalamus and is activated by light. Peripheral clocks oscillate in virtually all organs, including the gut, as all cell types are synchronized and entrained by autonomic innervation and humoral factors. The core molecular clock consists of transcriptional and translational feedback loops. The clock proteins BMAL1 and CLOCK form a heterodimer and induce the expression of other clock proteins by binding to E-box motifs. These include BMAL1 positive regulators, such as RORα, and negative regulators, such as REV-ERBs, PERs and CRYs, which simultaneously downregulate their own transcription and initiate a new transcription cycle. DBP and NFIL3 form another loop that regulates the transcription of genes containing D-box sequences, including those for PER, and thus work in concert with the core clock to establish robust 24-hour rhythms. In inflammatory bowel disease (IBD), clock proteins interact with inflammatory mediators. CLOCK positively regulates and REV-ERBα, RORα or BMAL1 negatively regulate the expression of the transcription factor NF-κB, which is responsible for the activation of a variety of mediators involved in inflammation, such as the pro-inflammatory cytokines TNF-α, IL-1β or IL-6. NF-κB, in turn, inhibits the expression of E-Box-regulated clock proteins and thus may generally reduce clock gene expression during the inflammatory process as seen in IBD. Figure partly created with Biorender.com.

The molecular core of the clock consists of transcriptional and translational feedback loops (Figure 1) [3,4]. Specifically, two core clock proteins—aryl hydrocarbon receptor nuclear translocator like (ARNTL), also known as BMAL1, and circadian locomotor output cycles kaput (CLOCK) (paralogous to NPAS2)—activate clock gene expression by binding to E-box motifs [3]. To this end, CLOCK and BMAL1 form a heterodimer complex that enters the nucleus and induces the expression of genes encoding cryptochromes (CRY) and periods (PER) by binding to E-box sequences in their promoters [4]. As a result, PER and CRY are released into the cytoplasm. They bind together and re-enter the nucleus as a new complex. Once inside the nucleus, they interfere with the BMAL1:CLOCK complex and stop their own transcription [3,4,5].

In a second transcriptional/translational feedback loop, BMAL1:CLOCK drives expression of genes encoding REV-ERBα (also known as NR1D1) and RORα [3,4]. These proteins compete for the retinoic acid-related orphan receptor (ROR)-binding elements (RORE). While RORα binding induces *BMAL1* expression, REV-ERB binding inhibits *BMAL1* expression [3]. However, little is known about how *CLOCK* is regulated [2,6,7,8].

Another loop involves D-box-binding protein (DBP). DBP is also regulated by the BMAL1:CLOCK complex [3]. Together with interleukin-3-regulated protein (NFIL3), it forms a complex that binds to the D-box elements. These elements regulate genes containing D-box sequences, including those for PERs and RORs [3].

In addition to the pacemaker in the SCN, autonomous clocks are also present in peripheral tissues [9,10,11]. Their clock gene expression is locally controlled and independent of the SCN. However, they are entrained by the SCN through neuronal and hormonal pathways [10]. The endocrine system is central to the synchronizing of SCN and peripheral clocks [11]. It is thought that diet-related hormones synchronize the peripheral clocks in metabolic organs, the kidney, the gastrointestinal tract and the muscles [11].

Clock changes have been observed associated with jet lag and shift work [12,13] or stress [14]. Diet influences the transcription factors DBP and NFIL3, which have an effect on the length of the clock cycle [15]. In addition, environmental influences such as cigarette smoke have been shown to adversely affect circadian pathways [16]. Because of its vital role in the maintenance of the body’s homeostasis, the circadian clock is critical to disease mechanisms. Disruption of the clock has been implicated in diseases such as cardiovascular disease [17], obesity [18], diabetes [19,20], metabolic diseases [21], and cancer [22,23,24]. Furthermore, inflammatory bowel disease (IBD) and colorectal cancer have been linked to a dysfunction of the biological clock [25,26,27].

### 1.2. Inflammatory Bowel Disease (IBD)

IBD is a group of chronic inflammatory diseases affecting the gastrointestinal tract. Crohn’s disease (CD) and ulcerative colitis (UC) are the most common [28,29]. While CD spreads throughout the entire tract, UC primarily affects the large intestine, the colon and the rectum. While CD may impair several tissue levels, UC afflicts only the first inner layer of the colon [28,30]. Common symptoms include diarrhea and abdominal pain, while rectal bleeding is more than twice as likely to indicate UC as CD [31,32]. Other indicators are decreased appetite, weight loss, fatigue, anemia, joint pain, menstrual irregularity or cessation and fever [30].

In recent years, the prevalence of IBD has been highest in Western countries. In 2010, approximately 1.5 million cases were reported in the United States [33]. Ten years later, in 2020, this number increased by more than 60% to nearly 2.5 million cases [33]. Worldwide, the incidence of the disease is increasing, especially in Asia [34,35] and in emerging countries [34]. In Europe, the mortality rate in patients with CD is up to 40% higher than in the general population [36]. Incidence rates vary between different ethnic groups [37,38] and regions [37].

IBD is diagnosed by blood and stool tests, endoscopy, radiology scans, CT scans or magnetic resonance imaging [39,40]. It has been suggested that multiple factors [32], including environmental exposures, such as smoking or drugs [41,42], as well as diet [43,44,45], exercise [46,47], genetics [37,38], sleep [48,49] and depression [50,51] contribute to the development of IBD. IBD has also been associated with disturbed circadian rhythms, such as altered sleeping [52,53,54,55] and eating [56] habits. 

This review provides an overview of study results on the relationship between the circadian clock and IBD. The study results suggest that IBD and biological clocks influence each other and thus open up new therapeutic perspectives.

## 2. Results

### 2.1. Influence of IBD on the Circadian Rhythm

Significant clock gene disruption was found in both CD and UC cases. The expression of the clock genes *BMAL1, CLOCK, PER1/2* and *CRY1/2* was up to three times lower in the mucosal tissue and peripheral blood of IBD patients [57,58]. Comparing CD and UC, UC patients had significantly lower *PER1/2* and *BMAL1* expression in inflamed mucosa than CD patients [57]. No differences in clock gene expression between UC and CD patients were found in peripheral blood mononuclear cells [57,58].

In addition to clock gene alterations, IBD patients showed upregulated genes involved in cell differentiation (*BHLHE40*, *BHLHE41*) [59] and downregulated genes involved in cell growth (*KITLG*, *EGFR*, *EREG*) [59]. Correlations between clock gene expression and Mayo score, an index of colitis activity, were predominantly negative [57]. In addition, the levels of two inflammation markers, C-reactive protein and calprotectin, were significantly increased in IBD patients compared with controls [58]. Table 1 summarizes the association between IBD and clock gene changes in humans. One study, did not differentiate between UC and CD [60].

Clock gene expression associated with intestinal inflammation has also been studied in mice [57,62,63,64]. In these studies, colitis was induced by treatment with dextran sulfate sodium (DSS) or 2,4,6-trinitrobenzene sulfonic acid (TNBS). Table 2 summarizes the changes in clock gene expression in mice with colitis. 

Examination of C57BL/6 mice with DSS-induced colitis revealed significantly reduced *Cry1*, *Per2*, *Npas2* and *Rev-erbα* expression, but significantly increased expression of *Rorα* [63]. In contrast, another study reported no changes for *Bmal1*, *Clock* and *Rev-erbα* expression in response to DSS treatment [62]. In addition, some DSS-treated mice received ocular treatment with UVB light (range 280–320 nm) for 60 s each day. DSS + UVB light-treated mice showed significantly increased *Bmal1*-, *Clock*- and *Rev-erbα*-mRNA levels and even higher colitis scores than the DSS-only and control groups. Accordingly, UVB eye irradiation exacerbates the effects of colitis [62].

### 2.2. Influence of the Circadian Rhythm on Colitis

The effects of IBD on clock gene expression were not unidirectional. Clock disruption also affected the disease, highlighting a bidirectional relationship. Mice with inactivated or completely knocked out clock genes showed changes in cells, genes and colitis, as summarized in Table 3. Mice lacking *Rorα* or *Bmal1*-driven *Lnc-UC* were more susceptible to colitis than their control group [65,66]. *Lnc-UC* is a long noncoding RNA that has been associated with colitis in mice and humans, particularly by reducing *Rev-erbα* expression [66]. On the other hand, healthy *Lnc-UC* levels along with high *Rev-erbα* levels reduced colitis. *Lnc-UC* deactivated the activity of NLR family pyrin domain (NLRP) 3, an inflammasome critical for the induction of proinflammatory cytokines [63,66]. Mice lacking *Nlrp3* did not respond to DSS-induced colitis [63].

Mice with clock disruption caused by jet lag or *Bmal1* deficiency were more susceptible to DSS treatment [57,67]. Shifts in the light–dark cycle caused greater damage from DSS [57]. Similarly, disease activity scores of mice with DSS-induced colitis worsened after sleep deprivation [60]. Furthermore, DSS-induced colitis was more severe in *Per1/2*-deficient mice compared to wild-type mice [68]. While 30% of the epithelium of *Bmal1^−/−^* mice was damaged by DSS, only half of the damage was observed in the wild-type group [67]. The degree of inflammation also depended on *Rev-erbα* expression as *Rev-erbα* inhibited nuclear factor (NF) κB signaling and NLRP3. Whenever *Rev-erbα* expression was elevated due to its circadian behavior, the severity of inflammation was reduced [66]. 

Moreover, colitis damage was found to be time-dependent [66]. *Bmal1^−/−^* mice showed a consistently high inflammation rate, whereas inflammatory activity in *Bmal1^+/+^* mice varied throughout the day. This suggests that clock-deprived animals are more susceptible to colitis damage and that disease activity varies throughout the day in mice with functioning clock rhythms [64]. DSS mice under jet lag had increased colitis damage compared to mice treated with DSS alone [69].

A critical part of the development of IBD is the stability and function of the intestinal barrier [70,71,72,73]. Tight junctions close gaps between epithelial cells to prevent the passage of inflammatory substances. The tight junction proteins occludin and claudin-1 showed circadian oscillation opposite to *Per2* mRNA [74]. While mice that lacked PER2 had persistently elevated levels of occludin and claudin-1, mice lacking CLOCK had persistently low levels of the two tight junction proteins and were more susceptible to intestinal injury from DSS [74]. Therefore, it has been suggested that colonic permeability is clock-dependent, with opposing functions of CLOCK and PER2 in the clock rhythm [74]. In contrast, no differences regarding the transcriptional expression of occludin, claudin-1 and two other barrier genes (tight junction protein 1 and mucin 2) were found between colitis mice lacking *Bmal1* and their nonmutant controls [64].

**Table 3:** Effects of clock disruption on cells, genes and colitis in mice. * *p* < 0.05. If no comparison group is given compared to controls. ↑ = significantly upregulated levels; ↓ = significantly downregulated levels; JL = jet lag; DSS = dextran sulfate sodium; TNBS = 2,4,6-trinitrobenzene sulfonic acid; IEL = intraepithelial lymphocytes; ⇔ = stable levels; DAI = disease activity index score; SD = sleep deprivation.

Reference Year	Samples	Disrupted Clock Mouse Models	Additional Treatment	Findings
[74] 2014	Colon tissue	*mPer2^m/m^*		occludin↑*, claudin-1↑*
			DSS	increased DSS resistance
		*Clock* ^Δ*19/*Δ*19*^		occludin↓*, claudin-1↓*
			DSS	increased DSS sensitivity
[57] 2017	Colon tissue	JL	DSS/TNBS	increased DSS/TNBS sensitivity and damage
[68] 2017	Colon and ileal tissue	*Per1/2^−/−^*		paneth cell↓*, goblet cell↓*, lysozyme transcript/protein↓* inhibited cell proliferation and apoptosis
			DSS	mucin 2↓* increased DSS sensitivity
[63] 2018	Colon tissue	JL		*Rev-erbα*↓*
			DSS	increased DSS sensitivity
		*Bmal1^−/−^*		*Rev-erbα*↓*
			DSS	increased DSS sensitivity
		*Rev-erbα^−/−^*	DSS	increased DSS severity REV-ERBα inactivates NLRP3
[65] 2019	Colon tissue	*Rorα* ^Δ*IEC*^	DSS	Ki67+↓*, 16S rDNA↑* RORα essential for recovery RORα reduced NF-κB transcription
[66] 2020	Colon tissue	*Bmal1^−/−^*		*Lnc-UC*↓*, *Dbp*↓*
		*Lnc-UC* ^−/−^	LPS	*Rev-erbα*↓* *Lnc-UC* regulated inflammation
[67] 2021	Intraepithelial lymphocytes	*Bmal1^−/−^*	DSS	IELs Breg Cells↓*
[64] 2022	Colon tissue	*Bmal1^−/−^*	DSS	NR1D1↓*, claudin-1⇔, mucin 2 ⇔ poor regeneration
[69] 2022	Colon tissue	JL		*Per2*↓*, Ki67↓*, all core clock genes disrupted destroyed mitochondrial morphology
		JL	DSS	increased DAI
[60] 2022	Colon tissue	SD	DSS	*Cry2*↑* increased DAI

However, mucin 2 mRNA, a gut-protective secretory protein [75], was significantly reduced in DSS-treated mice lacking *Per1/2* compared with DSS-treated wild-type mice [68]. Likewise, significantly reduced numbers of secretory Paneth cells, goblet cells and lysozyme were detected in colitis-affected mice without *Per1/2* [68]. In contrast to the secretory cells, the total number of epithelial cells in the colon, ileum and stem cells in the small intestine hardly changed [68]. No changes in goblet cells or crypt abscess scores were found in *Bmal1*-deficient colitis mice [68]. Nevertheless, *Bmal1^−/−^* mutants showed significant morphological abnormalities and increased immune cell infiltration compared to wild-type colitis mice [64]. In addition to alterations in intestinal barrier cells, DSS-injured mice lacking the circadian transcription factor RORα showed a twofold increase in bacterial 16S rDNA in the mesenteric lymph node after 14 days, indicating increased intestinal barrier permeability [65].

In addition to gut barrier function, circadian rhythmicity has also been linked to the immune system [67,68]. Under the influence of DSS/TNBS and time shift, mice showed significantly higher levels of inflammatory cytokines interleukin (IL) 6 and tumor necrosis factor (TNF) α than without time shift [57]. Influenced by BMAL1, TNF-α was expressed more strongly in *Bmal1^−/−^* mice [64]. Furthermore, disruption of the biological clock resulted in a reduction of regulatory B cells, a subset of B cells that suppress the immune system and increase immunological tolerance [67]. Tissue-dependent changes in T cells, lymphocytes, natural killer cells and dendritic cells were also reduced. Therefore, a BMAL1-driven B cell regulation has been proposed [67].

Finally, differences in heterozygous and homozygous variants of the clock gene *PER3* have been observed in IBD patients [61]. Significant genotype differences were found when comparing the adult-onset group with the control group [61]. One allele was determined to be more frequent in the adult-onset group than in the control group. This “risk” allele was present in two out of three genotypes. It was concluded that IBD correlates with the polymorphism of *PER3* [61].

### 2.3. Circadian Rhythmicity and Cell Proliferation

Continuous processes of regeneration and cell growth are necessary to recover from cell damage and inflammation [76]. These processes are preventative and follow circadian rhythms [77]. Cell proliferation was impaired 24 h after injection of a proliferation marker into *Per1/2*-deficient mice [68]. WEE1, an inhibitor protein kinase that regulates mitosis, increased mRNA levels in clock gene-deficient mice and impeded cell division, which was associated with decreased cell apoptosis but increased necroptosis [68].

It was found that regulatory B cells expressing high levels of programmed cell death ligand 1 (PDL1) were regulated by BMAL1 [67]. When these cells were injected into the blood of mice with clock disorders, these mice were less affected by colitis. Conversely, the absence of PDL1^+^ B cells promoted colorectal cancer through CD4^+^ T cell apoptosis [67]. In contrast to *Bmal1^−/−^* mice with colitis, in which epithelial cell proliferation was consistently low, cell proliferation in diseased *Bmal1^+/+^* mice fluctuated throughout the day [64]. Less than 20 % of *Bmal1^−/−^* mice recovered from their DSS treatment compared to more than 75% of *Bmal1^+/+^* mice [64].

In jet-lagged mice, Ki67, a cell proliferation marker, and p-DRP1, a molecule active in ATP production, as well as ATP itself showed reduced expression [65]. Cell proliferation was also reduced in *Per2-silenced* mice. Thus, chronodisruption impaired mitochondrial shape and function, which reduced ATP production. Since ATP production was linked to cell proliferation, cell proliferation was also reduced [69]. RORα has been linked to p65, a major subunit of NF-κB, which is also essential for the recovery process of inflamed tissues [65]. It has been suggested that RORα competes with two other proteins, CREB-binding protein and Bromodomain-containing protein 4, to reduce NF-κB transcription [65]. While wild-type mice were able to recruit RORα, intestinal epithelial cell-specific RORα-deficient (RORα^ΔIEC^) mice were unable to recruit RORα, resulting in intense inflammation despite the recovery period. Therefore, it was found that RORα is necessary to recover from colitis and maintain a functional intestine by reducing NF-κB transcription [65].

## 3. Discussion

We summarized recent findings on the relationship between IBD and the circadian clock on a molecular basis. It has become clear that the circadian rhythm affects the onset, severity and recovery of IBD. At the same time, intestinal inflammation has been reported to disrupt the biological clock. Previous analyses of the biological clock and IBD found strong relationships between the circadian clock, intestinal defense and the immune system [73,78].

The proinflammatory markers TNF-α and IL-1β were found to control epithelial barrier function [71]. Colitis decreased cell proliferation [64,68] and impaired the development of physiological function. Furthermore, inflammation led to the loss of several secretory cells [68], which play an important role in the immune response. Disruption of clock genes could also reduce the number of secretory cells [68]. Therefore, as previously suggested, clock disruption may create an imbalance in gut physiology while inflammation disintegrates it [68]. The impact of the circadian clock on inflammation extends beyond the intestine. Inflammatory arthritis [79,80], neuroinflammation [81,82] and metabolic inflammation [83] have been linked to the biological clock. In addition, inflammation of the retina [84], skin [85] and lungs [86,87,88] has been linked to circadian rhythms.

Immune cells exhibit circadian behavior [89,90] and clock genes have been found to directly influence innate immunity. In *Rev-erbα^−/−^* mice, lipopolysaccharide (LPS)-induced inflammation was worse than in wild-type mice. Increased translocation of p65, a subunit of the heterodimer NF-κB protein complex [91], into the nucleus was found, indicating an upregulated NF-κB pathway [81]. NF-κB regulates inflammation through transcription of proinflammatory cytokines and other molecules [92]. It is activated in response to stimulation by bacterial and viral factors via pattern-recognition receptors, such as Toll-like receptors (TLR), or cytokines like TNF-α [91,93]. In *Rev-erbα^−/−^* mice, several genes involved in the positive regulation of NF-κB signaling, such as *Nfkb2*, *Tlr4*, *Stat3* and *Traf2*, were found to be upregulated [81]. Two genes involved in inhibiting NF-κB signaling (*Nfkbib* and *Usp31*) were found to be downregulated [81]. In primary microglia, *Rev-erbα*-binding peaks were localized at *Bmal1* promoters, known to be the target of *Rev-erbα*, but also at NF-κB promoters [81]. Therefore, a direct control of NF-κB by *Rev-erbα* has been proposed [81].

Notably, the NF-κB subunit p65 was directly linked to CLOCK, as CLOCK was found to coimmunoprecipitate with p65 [91]. In addition, CLOCK increased p65-mediated transcription in a concentration-dependent but BMAL1-independent manner [91]. In transcriptional assays of a κB-responsive promoter, activation was higher for plasmids with CLOCK/p65 co-expression than for plasmids with CLOCK/BMAL1/p65 co-expression [91]. Therefore, it has been suggested that BMAL1 opposes CLOCK/p65 co-activity [91]. In mice with reduced *Clock* expression (*Clock^+/−^* mice), activation of NF-κB by the TLR 5 agonist CBLB502 was also reduced, suggesting that CLOCK can increase the activity of NF-κB promoters [91]. Furthermore, CLOCK overexpression correlated with increased p65 acetylation, indicating p65 activity [91]. Thus, it has been proposed that CLOCK enhances NF-κB transcriptional activity by activating p65 [91]. However, the specific molecular mechanisms between CLOCK and NF-κB remain unclear [91].

As a result of NF-κB stimulation, the expression of repressive clock genes of the feedback loop was reduced [94]. The p65 peak density was found to be adjacent to E-box elements, indicating a disruption of the repressive side of the circadian clock [94]. p65 bound to the promoter sites of *Per1/2*, *Cry2*, *Dbp* and *Rev-erbα*. Consistently, their expression increased after *p65* knockout [94]. Deletion of the NF-κB regulatory kinase *IKKβ* led to similar results with a fold increase in repressor arm clock genes, indicating the regulatory influence of NF-κB in addition to *IKKβ* on clock rhythmicity [94]. Regulator regions of *Bmal1* and *Clock* were barely affected by p65 binding [94]. 

Interestingly, LPS-mediated inflammation in wild-type mice resulted in new CLOCK- and BMAL1-binding sites compared to saline-treated wild-type mice [94]. These sites were located near genes involved in immune response and metabolic signaling pathways, among others [94]. Therefore, a circadian control of inflammatory response through epigenetic modulation has been proposed [94]. 

This interpretation was supported by findings in macrophages showing that the epigenetic states of enhancers were regulated by BMAL1 [95]. BMAL1 bound to the clock gene loci in primary macrophages of mice, but also to TLR-4 inducible genes (*Hif1a* and Csf1r) [95]. Moreover, the aforementioned novel CLOCK-/BMAL-binding sites due to LPS inflammation showed increased NF-κB-binding motifs [94]. CLOCK/BMAL1 shifted to sites associated with increased p65 binding [94]. Therefore, it has been suggested that CLOCK/BMAL1/p65 colocalization is dependent on LPS-induced inflammation [94]. In conclusion, increased NF-κB activity suppresses the transcription of repressor arm clock genes and the relocalization of activator arm clock proteins. Consequently, increased NF-κB activity alters circadian clock transcription processes (see Figure 1).

Besides its influence on the NF-κB pathway, the biological clock has been linked to other parts of the immune response. Among those, influences on cytokine IL-1β were found [96]. In *Bmal1^−/−^* mice, IL-1β-production was higher than in *Bmal1^+/+^* mice. Knockdown of *Nfr2*, an inhibitor of IL-1β, led to similar results [97]. In contrast, NFR2 activation was enhanced by BMAL1 binding to the E-box in the *Nrf2* promoter [96]. Consequently, the immune response via IL-1β was suppressed and inflammation was alleviated. BMAL1 regulatory effects on inflammation have also been linked to REV-ERBα [85]. In *Bmal1* knockout mice with skin inflammation, p65 and NLRP3 levels were increased compared to their wild-type counterpart [85]. *Rev-erbα^−/−^* mice were more sensitive to skin inflammation associated with the direct effects of clock genes on NLRP3 [63,85]. Furthermore, silencing of *Rev-erbα* attenuated the regulatory influence of BMAL1 on inflammation, suggesting that regulation by BMAL1 occurs via REV-ERBα [85]. As a mediator of inflammatory infiltration of macrophages [98], REV-ERBα has also been proposed as a critical link between the circadian clock and adaptive immune responses [99]. T helper 17 (Th17) cells enhance the immune response by producing the cytokine IL-17 [100]. RORα and RORγt regulate them. REV-ERBα competes with RORγt by binding to the RORE of Th17, thereby inhibiting the Th17 immune response [101,102,103].

BMAL1 deficiency was also related to increased levels of chemokines, which signal inflammatory processes and attract monocytes [103]. The secretion of cytokines, such as TNF-α- and IL-6, showed a circadian rhythmicity that followed the diurnal rhythms in peripheral organs [104]. The circadian clock was involved in TNF-α transcription upon TLR4 signaling in response to LPS stimulation [104]. Interestingly, TNF-α and IL-1β disrupted the circadian rhythm [105,106]. However, only clock genes that depend on E-box-mediated transcription were affected [105]. Besides cytokines, many immune cells, such as natural killer cells, lymphocytes and neutrophils, underlie circadian rhythmicity [107]. Reduced clock gene expression in natural killer cells affected the rhythmicity of cytolytic factor secretion [108]. 

In addition, the microbiota influenced the rhythmicity of the immune system [109], which showed diurnal oscillations [110,111]. A comparison of *Bmal1^IEC^^−/−^* mice showed that the gut clock system also drives microbiota oscillations. Microbial functionality was altered when intestinal rhythmicity was disabled [112] and specific populations, such as Bacteroidetes *S24-7* spp. and *Prevotella*, Firminicutes *Allobaculum* and *Lactobacillaceae* spp. and Protobacteria *Heliobacter* and *Suterella,* were reduced [113]. Microbial rhythmicity was completely lost in *Per1/2^−/−^* mice [110]. Circadian disruption by jet lag also disrupted diurnal oscillations in the microbiota of mice [110]. However, jet lag was not always sufficient to disrupt the microbiome in mice, but a combination of jet lag and a modified high-fat, high-sugar diet altered the microbiome [111].

Interestingly, microbiota and circadian rhythms show bidirectional behavior. The regulation of the circadian transcription factor nuclear factor interleukin 3 (Nfil3) was dependent on the microbiota, which could repress the expression of *Rev-erbα* [114]. In general, microbiota depletion was associated with changes in clock gene expression [115]. Mice with depleted microbiota had a disrupted core clock. While *Bmal1* and *Cry1* were reduced, *Per1/2* increased and *Clock* was unaffected [116].

Besides its impact on the circadian rhythm, the microbiota plays a central role in the pathogenesis of IBD. In most mouse models, the microbiota is required for the development of intestinal inflammation [117]. Caspase-3-deficient mice that were initially safe from colitis could develop colitis when housed with wild-type mice [118]. Therefore, microbiota transfer through cohousing has been implicated as a reason for the disease development [118]. Furthermore, some bacteria have been shown to be destructive to the barrier and lead to inflammation [119], while others have been shown to aid in the development of the immune system and intestinal defense [120,121]. Antimicrobial peptides synthesized by gut bacteria help protect the intestinal barrier from damage [122].

Several limitations were identified in the reviewed studies. In the mouse studies, the method of chemically inducing colitis varied in amount and timing, which could affect disease severity. Additionally, the jet lag phases used to induce clock disruption differed in duration and number of phases [57,63,69]. For example, clock gene expression levels in mice were measured every fourth hour [63,66,69], every sixth hour [74] or every eighth hour [64]. Standardized times, intervals and methods to induce colitis or clock disruption would improve the comparability of studies. Although various clock genes and mechanisms have been studied in mice, comparable analyses in humans are lacking. Therefore, clinical trials in humans should support findings from animal studies.

In most human studies, the timing of tissue collection was not specified [58,59,60,61]. Therefore, a standardized approach to tissue collection and clock gene expression analysis is required. Clinical trials involving human participants should consider establishing more specific guidelines regarding sleep and mealtimes. Comparable circadian time points could help to increase the value of clock gene expression measurements. As circadian rhythmicity may still vary between human individuals, circadian markers like melatonin could help to validate results.

Moreover, studies evaluating the long-term relationship between IBD and clock genes are needed. Cohort studies of changes in disease activity and symptoms associated with several different rhythm-related factors, such as sleep–wake patterns, meal timing and light exposure, may help to understand the impact of each factor. At the same time, more specific claims could be made about the entrainment of peripheral clocks. 

Today, IBD is treated medically or surgically. Five types of medications dominate the treatment of IBD [39,123]. First, aminosalicylates reduce inflammation and inhibit symptoms. Second, corticosteroids regulate and suppress the immune system. Immune modulators prevent continuous inflammation due to the relentless activity of the immune system. Fourth, antibiotics are used to cure colorectal infections. Finally, biological therapies aim to prevent tumor necrosis factors and white blood cells from entering IBD-affected tissue [39,123,124].

Recent studies recommended fecal microbiota transplantation as a promising and effective treatment for UC [125,126]. In addition, dietary modification has been suggested to be beneficial in the future treatment of IBD [127]. Similarly, melatonin has shown positive effects in reversing the disease [128]. Beyond melatonin, drugs that target specific clock genes are opening up a new field of research. Furthermore, the timing of medication, dietary intake or sun-/screen light exposure should be more emphasized in the treatment process. More specifically, taking the biological clock into account should be part of personalized medicine, which aims to include the time factor as an important part of therapy. 

Next to regular sleeping times, adequate rest and regular mealtimes, the disease progression may benefit from limiting factors that are detrimental to IBD and the circadian clock. First and foremost, abstinence from smoking and alcohol should be considered. Besides physical changes, the patient’s mental state may need to be evaluated and treated. 

## 4. Conclusions

The biological clock has received increasing attention and has been implicated not only in various physiological functions and processes, but also in diseases such as intestinal diseases, especially IBD. Several studies have addressed the relationship between IBD and the biological clock. They unanimously link the clock and disease mechanisms. Therefore, the molecular link between the biological clock machinery and IBD should be considered as a potential future therapeutic target. Although a connection between NF-κB and the circadian rhythm has been established, the understanding of the specific mechanisms remains incomplete. Moreover, information on the interplay between inflammation and the biological clock beyond the NF-κB pathway is fragmentary. It remains to be seen how exactly the biological clock and IBD are linked at the molecular level and how existing knowledge about the biological clock can be used to effectively treat the disease.

## 5. Methods

A literature search was conducted for articles on the biological clock and IBD using search terms “clock” and “circadian” for biological clock and “intestine”, “gut”, “inflammatory bowel disease”, “ulcerative colitis”, “Crohn’s disease” and “colitis” for IBD. Besides these terms, titles and abstracts were scanned for clock-related terms, e.g., “circadian rhythm” or specific clock genes like PER2. Citation tracking of valid study documents was performed. Four databases were searched for potential research articles: PubMed, Google Scholar, Clinical Trials and Cochrane. The search was conducted until December 2022. In total, titles and abstracts of 759 articles were scanned, and 28 articles were assessed for eligibility. The search resulted in 14 articles (see Supplementary Figure S1).

## Figures and Tables

**Table 1:** IBD and clock gene expression in humans. * *p* < 0.05 compared to healthy controls. † *p* < 0.05 inflamed vs. noninflamed tissue. ↑ = significantly upregulated mRNA levels; ↓ = significantly downregulated mRNA levels; CD = Crohn’s disease; UC = ulcerative colitis; IBD = inflammatory bowel disease; CGE = clock gene expression; CRP = C-reactive protein; nuc = nuclear.

Reference Year	No. of Patients, Disease	Samples	Disease	Findings
[61] 2012	*n* = 3365 (972 UC, 1082 CD, 1311 HC)	Peripheral blood leukocytes	UC and CD	*PER3*-risk-variant↑*
[59] 2015	*n* = 29 (14 UC, 15 CD)	Inflamed or adjacent noninflamed colon tissue	UC and CD	*ARNTL2*↑*, *RORα*↑*, *PER3*↓*
			UC	*CRY1*↑*, *CSNK1E*↑*, *TIPIN*↑*, *NR1D2*↓*
			CD	*CSNK2B*↓*, *NPAS2*↓*, *PER1*↓*
[57] 2017	*n* = 132 (51 UC, 39 CD, 42 HC)	Inflamed colon mucosa	UC and CD	*BMAL1*↓*, *CLOCK*↓*, *PER2*↓*, *CRY1/2*↓*
			UC vs. CD	*PER1/2*↓*, *BMAL1*↓*
		PBMCs	UC and CD	*BMAL1*↓*, *CLOCK*↓*, *PER1/2*↓*, *CRY1/2*↓* CGE correlated negatively with CRP
[58] 2020	*n* = 30 (5 UC, 8 CD, 16 HC)	Peripheral blood leucocytes	UC and CD	*BMAL1*↓*, *CLOCK*↓*, *PER1*↓*, *CRY2*↓* CGE correlated negatively with fecal calprotectin
		Colon mucosa	UC and CD	*CLOCK*↓^†^*, PER1*↓^†^*, CRY2*↓^†^
[60] 2022	*n* = 103 (IBD)	Colon mucosa	UC and CD	*BMAL1*↓^†^, *CRY1/2*↓^†^, *REV-ERBα*↓^†^ sleep quality correlated with disease severity

**Table 2:** Colitis’ effect on Clock gene expression in colitis mice. * *p* < 0.05 compared to controls. ^††^ *p* < 0.05 compared to DSS-only group. ↑ = significantly upregulated mRNA levels; ↓ = significantly downregulated mRNA levels; DSS = dextran sulfate sodium; TNBS = 2,4,6-trinitrobenzene sulfonic acid; UVB = UVB eye irradiation.

Reference Year	Samples	Colitis Mouse Models	Findings
[57] 2017	Colon mucosa	DSS/TNBS	Bmal1↓*, Clock↓*, Per1/2↑*, Cry1/2↓*
[62] 2018	Colon tissue	UVB+DSS	Clock↑^††^, Bmal1↑^††^, Rev-erbα↑^††^, Nfil3↓^††^, Rorγt↑^††^ UVB worsened DSS severity
[63] 2018	Colon tissue	DSS	Per2↓*, Cry1↓*, Rev-erbα↓*, Npas2↓*, Rorα↑* Clock, Rev-erbβ, Dbp disturbed severity of clock gene disturbance depends on daytime
[64] 2022	Colon tissue	DSS	reduced rhythm amplitude

## Data Availability

No new data were created or analyzed in this study. Data sharing is not applicable to this article.

## Supplementary Materials

The following supporting information can be downloaded at: https://www.mdpi.com/article/10.3390/clockssleep5020021/s1.
