# Supplementary material for: Relationship between the Biological Clock and Inflammatory Bowel Disease
Source: Clocks Sleep. 2023 May 12;5(2):260–75. doi: 10.3390/clockssleep5020021 (PMC10204364; doi:10.3390/clockssleep5020021)
Supplement: Supplementary file 1 [file clockssleep-05-00021-s001.zip › clockssleep-2286525-supplementary.pdf]

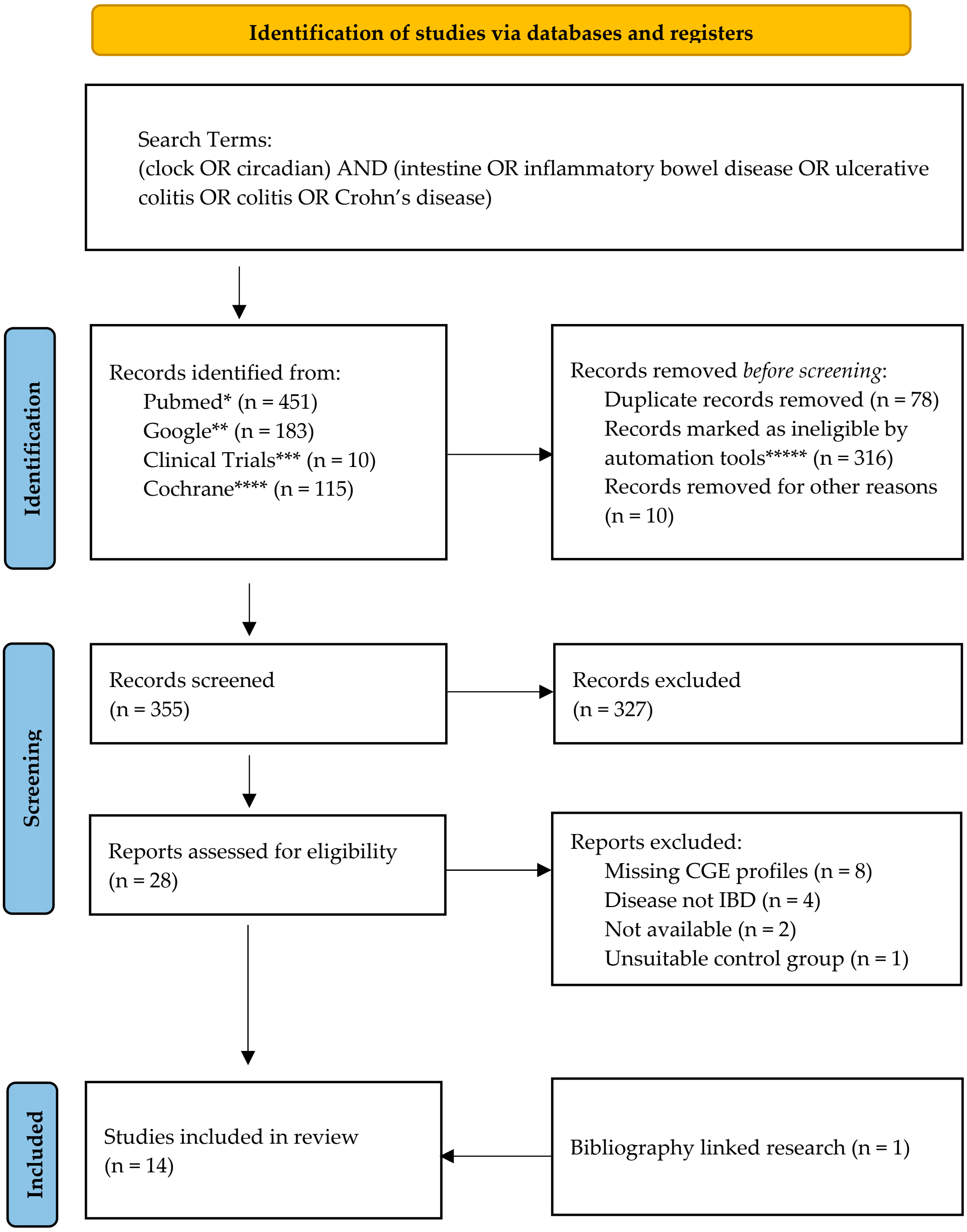

Figure S1: Literature Search

*(title, abstract) + search terms; exact query: (("clock"[Title/Abstract]) OR ("circadian"[Title/Abstract])) AND ((((("intestine"[Title/Abstract]) OR ("inflammatory bowel disease"[Title/Abstract]) OR ("ulcerative colitis"[Title/Abstract]) OR ("crohn s disease"[Title/Abstract]) OR ("colitis"[Title/Abstract]))))))

**title + search terms

***Disease: Inflammatory Bowel Diseases; Other terms: clock OR circadian

****Cochrane query: (title, abstract, keyword) + search terms

*****ineligibility criteria: research from the past century (beyond year 2000), review articles
